# Radial artery vasomotor function following transradial cardiac catheterisation

**DOI:** 10.1136/openhrt-2016-000443

**Published:** 2016-09-26

**Authors:** A J Mitchell, N L Mills, D E Newby, N L M Cruden

**Affiliations:** 1 Centre for Cardiovascular Science, University of Edinburgh, Edinburgh, UK; 2 Edinburgh Heart Centre, NHS Lothian, Edinburgh, UK

**Keywords:** Flow-mediated dilatation, Vascular injury, Transradial catheterisation

## Abstract

**Aims:**

To determine the reproducibility of flow-mediated dilation (FMD) and nitrate-mediated dilation (NMD) in the assessment of radial artery vasomotor function, and to examine the effect of transradial catheterisation on radial artery injury and recovery.

**Methods:**

Radial artery FMD and NMD were examined in 20 volunteers and 20 patients on four occasions (two visits at least 24 hours apart, with two assessments at each visit). In a further 10 patients, radial artery FMD was assessed in the catheterised arm prior to, at 24 hours and 3 months following cardiac catheterisation.

**Results:**

There were no differences in baseline radial artery diameter (2.7±0.4 mm vs 2.7±0.4 mm), FMD (13.4±6.4 vs 12.89±5.5%) or NMD (13.6±3.8% vs 10.1±4.3%) between healthy volunteers and patients (p>0.05 for all comparisons). Mean differences for within and between day FMD were 2.53% (95% CIs −15.5% to 20.5%) and −4.3% (−18.3% to 9.7%) in patients. Compared to baseline, radial artery FMD was impaired at 24 hours (8.7±4.1% vs 3.9±2.9%, p=0.015) but not 3 months (8.7±4.1% vs 6.2±4.4, p=0.34) following transradial catheterisation.

**Conclusions:**

Radial FMD is impaired early after transradial catheterisation but appears to recover by 3 months. While test–retest variability was demonstrated, our findings suggest that transradial access for cardiac catheterisation may afford a potential model of vascular injury and repair in vivo in man.

**Key questions**

**What is already known about this subject?**

- Radial artery vasomotor function is impaired following transradial catheterisation. However, the degree of this dysfunction and the ability of the artery to regain vasomotor function vary between studies.
- Radial flow-mediated dilation (FMD) is of a greater magnitude than brachial FMD and therefore potentially a more sensitive tool for examining the effects of cardiovascular risk factors and therapies. However, it has previously been shown to be a highly variable technique, limiting its widespread adoption.

**What does this study add?**

- We quantify the degree of radial FMD variability within and between days in healthy volunteers and patients. Transradial catheterisation rapidly suppresses radial artery function, but this appears to recover over a 3-month period.

**How might this impact on clinical practice?**

- Transradial catheterisation provides a useful in vivo clinical model of mechanical arterial injury and repair. This has major potential for application to experimental medicine studies and to assess the potential benefits of vascular regenerative and reparative therapies.

## Introduction

Difficulty in translating promising findings from preclinical models into the clinic^1^ is arguably exacerbated by a lack of human in vivo models to guide translational research and improve our understanding of human pathophysiology. An in vivo model to examine vascular injury and repair would afford mechanistic insights as well as the opportunity to test novel therapeutic approaches. While there are numerous animal models of atherosclerotic and mechanical injury,^2–8^ interspecies differences and imperfect replication of prevailing clinical conditions limit their direct translation to humans.

Although rarely resulting in clinically relevant sequelae, transradial catheterisation is associated with subclinical abnormalities of radial artery structure^9^ ^10^ and function^11–15^ as a consequence of the trauma of intraluminal sheath insertion. The routine use of transradial access in clinical care and the accessibility of the human radial artery to non-invasive imaging provide a unique opportunity to study the mechanisms of vascular injury and repair in vivo in humans.

Flow-mediated dilation (FMD) of the brachial artery is widely established as a tool to assess vasomotor function in vivo in man.^16^ While brachial artery FMD is well characterised in terms of reproducibility, radial FMD is not. This may limit its usefulness in this setting of a potential clinical experimental medicine model of arterial injury. The aim of this study was to determine the reproducibility of FMD and nitrate-mediated dilation (NMD) in the assessment of radial artery function, and to examine the temporal effect of transradial catheterisation on radial artery vasomotor function.

## Methods

### Participants

Twenty healthy volunteers and 30 patients undergoing diagnostic transradial cardiac catheterisation for stable angina were recruited. The project was approved by the Research Ethics Committee and written informed consent was obtained from all participants.

Participants were assessed at the same time of day and asked to avoid food for 4 hours and caffeine, vasoactive medications, smoking and alcohol for 24 hours prior study visits. All assessments were carried out in a quiet, temperature-controlled room and patients rested for 10 min before the first study measurements were made.

### Study protocols

#### Protocol 1

A total of 20 healthy volunteers and 20 patients attended on two occasions at least 24 hours apart. FMD and NMD of the left radial artery were assessed on two occasions per visit with at least 1 hour between repeat assessments.

#### Protocol 2

Ten patients undergoing elective cardiac catheterisation via the radial artery attended on three occasions (baseline and 24 hours and 3 months following cardiac catheterisation). FMD and NMD of the radial artery were assessed in the catheterised (right) arm at each visit.

### Flow-mediated and nitrate-mediated dilatation

FMD and NMD were carried out as per international guidelines.^16^ ^17^ Briefly, the radial artery was imaged 5 cm proximal to the radial styloid with a 12 MHz linear-array ultrasound transducer (CX50 Philips Amsterdam, the Netherlands) held in place by a stereotactic clamp. A baseline recording was captured over 60 s. A supra-systolic cuff was then inflated to 220 mm Hg for 5 min immediately distal to the antecubital fossa. Following release of the cuff, the artery was imaged continuously for 5 min (FMD). After 15 min of rest, the artery was once again imaged at rest for 60 s. Participants were then given 25 µg of sublingual nitrate and the radial artery imaged for a further 5 min (NMD). This process was repeated after a 1-hour rest period during which the participant was disconnected from the equipment and mobilised.

#### Systemic haemodynamics

Mean arterial pressure and heart rate were recorded during the resting period and after cuff release.

#### Image acquisition

Image acquisition was ECG-gated and arterial diameter captured during end diastole (R-wave triggered). The artery was initially identified using colour flow mapping. The probe position which gave the largest arterial diameter and clearest definition of the anterior vessel wall was chosen to minimise underestimation of lumen diameter. The focus position of the probe was set to the anterior vessel wall as this is the most challenging to resolve.^16^

#### Image analysis

Images were analysed offline using proprietary software (Brachial Analyzer, Vascular Tools, Medical Imaging Applications, Iowa City, Iowa, USA). Baseline and peak diameters were measured in millimetres with measurements averaged across 60 frames and 10 frames for baseline and peak values, respectively. FMD and NMD were expressed as percentage change in diameter from baseline.

### Statistical analysis

Results are reported as mean and SD unless otherwise stated, reproducibility data are reported as mean bias with 95% limits of agreement. Comparisons between groups were made using Student's t-test. Bland-Altman plots were used to examine reproducibility using data obtained from the left radial artery. Comparisons between time points in protocol 2 were made using one-way repeated measures analysis of variance (ANOVA). Statistical analyses were performed using SPSS V.21.0 (IBM Corp, Armonk, New York, USA).

## Results

### Participants

All participants (table 1) tolerated the procedures well. There was no change in mean heart rate or systolic blood pressure following FMD or NMD.

**Table 1:** Baseline characteristics of study populations

	Protocol 1		Protocol 2 Patients n=10
	Patients n=20	Healthy volunteers n=20	
Age	61±9	22±4	69±9
Female (%)	15 (75)	13 (65)	3 (30)
Heart rate	62±10	70±12	64±9
Systolic blood pressure	138±12	118±9	135±17
Smoker (%)	4 (20)	0 (0)	3 (30)
Coronary artery disease (%)	18 (90)	0 (0)	7 (70)
Diabetes (%)	4 (20)	0 (0)	2 (20)
Hypertension (%)	15 (75)	0 (0)	90 (90)
Hyperlipidaemia	15 (75)	0 (0)	70 (70)
Statin	20 (100)	–	9 (90)
Aspirin	19 (95)	–	10 (100)
β-Blocker	12 (60)	–	6 (60)
Nitrate	6 (30)	–	4 (40)
Calcium channel blocker	8 (40)	–	5 (50)
Baseline radial artery diameter (mm)	2.7±0.4	2.7±0.4	2.8±0.5

N (%), mean±SD.

### Flow- and nitrate-mediated dilatation

#### Arterial diameter

Baseline radial artery diameter was 2.7±0.4 and 2.7±0.4 mm in the 20 patients and 20 healthy volunteers, respectively. In cohort 2, there was no change in arterial diameter following catheterisation with measurements of 2.8±0.4, 2.83±0.37 and 2.82±0.39 mm at baseline, 24 hours and 3 months, respectively. Repeated measurements of baseline radial artery diameter demonstrated good reproducibility with intraday coefficient of repeatability (CR) of 0.35 and 0.45 and interday CR of 0.61 and 0.73 in patients and healthy volunteers, respectively.

#### Dilatation expressed as a percentage of baseline

**Figure 1:**
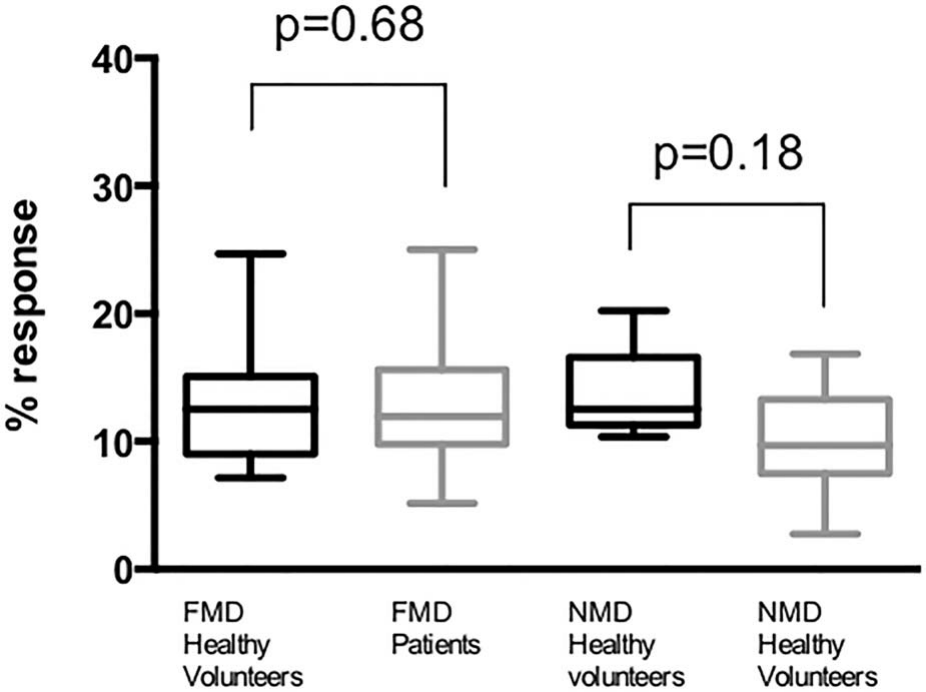
Baseline flow and nitrate mediated dilation in patients and healthy controls. Baseline flow-mediated dilation (FMD) response in healthy volunteers shows no difference from that seen in patients (13.4±6.4 vs 12.9±5.5%, p=0.68). Baseline nitrate-mediated dilation (NMD) response was also similar in healthy volunteers and patients (13.7±3.8% vs 10.2±4.4, respectively, p=0.18).

There were no differences in percentage FMD (13.4±6.4 vs 12.89±5.5%) or NMD (13.7±3.8 vs 10.2±4.4%) between healthy volunteers and patients, respectively (figure 1). There were no differences in the magnitude of response for repeat testing within or between days in either FMD or NMD (p>0.05 for all comparisons).

**Figure 2:**
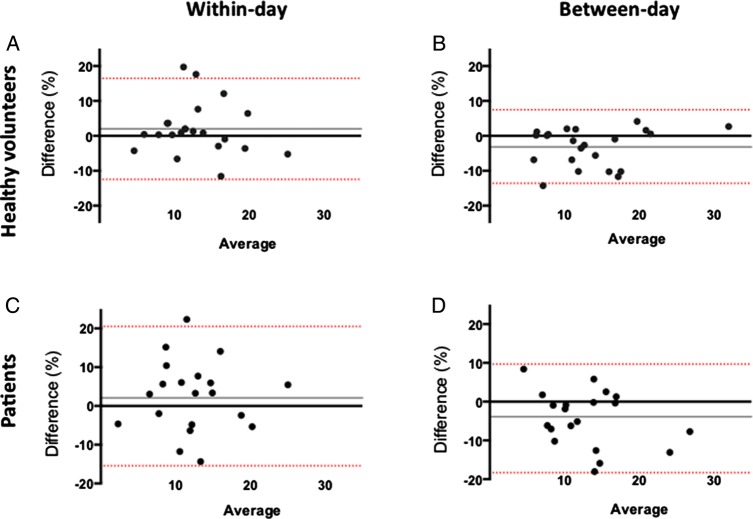
Reproducibility in percentage flow-mediated dilation. Within-day (A and C) and between-day (B and D) variability in healthy volunteer patients. The continuous grey line represents the mean of the differences of the two measurements (the mean bias) and the red lines represent the 95% CIs (limits of agreement).

**Figure 3:**
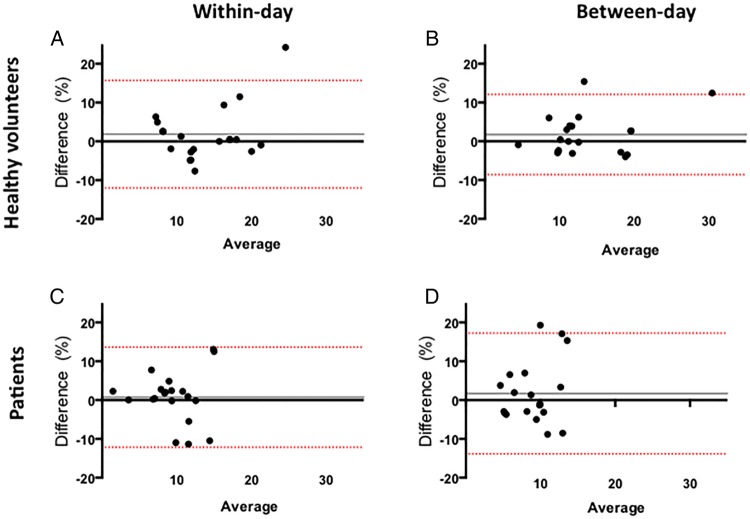
Reproducibility in percentage nitrate-mediated dilation. Within-day (A and C) and between-day (B and D) variability in healthy volunteer patients. The continuous grey line represents the mean of the differences of the two measurements (the mean bias) and the red lines represent the 95% CIs (limits of agreement).

In healthy volunteers, the mean of the differences for within-day and between-day measures (bias) was 1.99% (95% CIs −12.5% to 16.5%) and 3.2% (95% CI −13.8% to 7.5%), respectively, for FMD and 1.8% (95% CI −12.0% to 15.7%) and 1.7% (95% CI −8.7% to 12.1%), respectively, for NMD. In patients, the mean of the differences for within-day and between-day measures was 2.53% (95% CI −15.5% to 20.5%) and −4.3% (95% CI −18.3% to 9.7%), respectively, for FMD and 0.7% (95% CI −12.1% to 13.6%) and 1.7% (95% CI −13.9% to 17.3%), respectively, for NMD (figures 2 and 3).

#### Dilatation as absolute change in vessel diameter (mm)

There were no differences in absolute FMD (0.3±0.3 vs 0.2.±0.2 mm) or NMD (0.3±0.3 vs 0.2±0.2 mm) between healthy volunteers and patients, respectively. For FMD and NMD, there were no differences in the magnitude of responses for repeat testing within or between days (p>0.05 for all comparisons).

**Figure 4:**
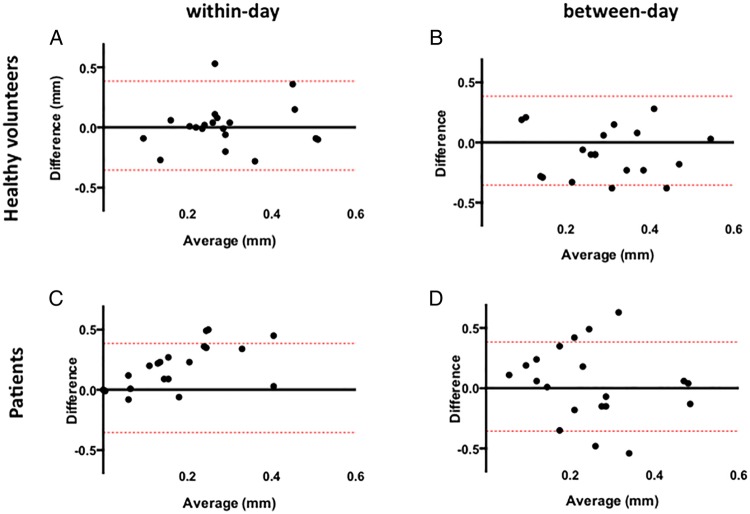
Reproducibility in absolute flow-mediated dilation. Within-day (A and C) and between-day (B and D) variability in healthy volunteer patients. The continuous grey line represents the mean of the differences of the two measurements (the mean bias) and the red lines represent the 95% CIs (limits of agreement).

**Figure 5:**
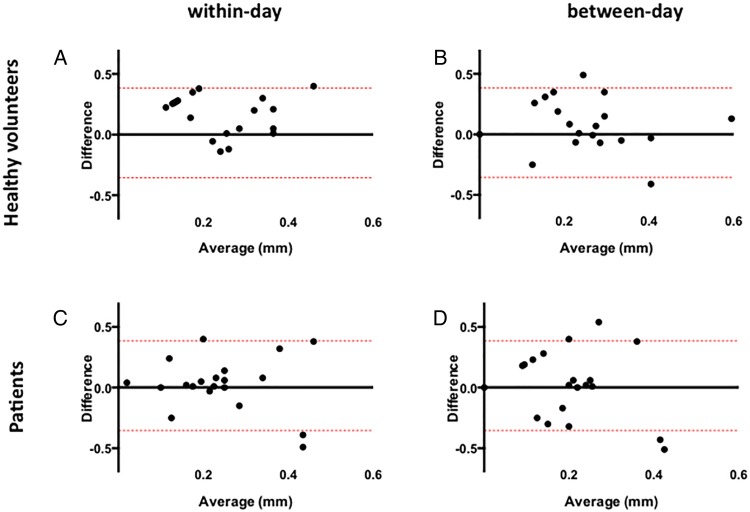
Variability in absolute nitrate-mediated dilation. Within-day (A and C) and between-day (B and D) variability in healthy volunteer patients. The continuous grey line represents the mean of the differences of the two measurements (the mean bias) and the red lines represent the 95% CIs (limits of agreement).

In healthy volunteers, the mean of the differences for within-day and between-day measures (bias) was −0.01 mm (95% CI −0.32 to 0.38 mm) and 0.03 mm (95% CI −0.3 to 0.31 mm), respectively, for FMD and 0.02 mm (95% CI −0.30 to 0.33 mm) and 0.03 mm (95% CI 0.35 to 0.38 mm), respectively, for NMD. In patients, the mean of the differences for within-day and between-day measures was 0.01 mm (95% CI −0.3 to 0.35 mm) and −0.02 mm (−0.35 to 0.38 mm), respectively, for FMD and 0.03 mm (95% CI −0.35 to 0.40 mm) and 0.02 mm (95% CI −0.30 to 0.35 mm), respectively, for NMD (figures 4 and 5).

#### Arterial function following catheterisation (cohort 2)

**Figure 6:**
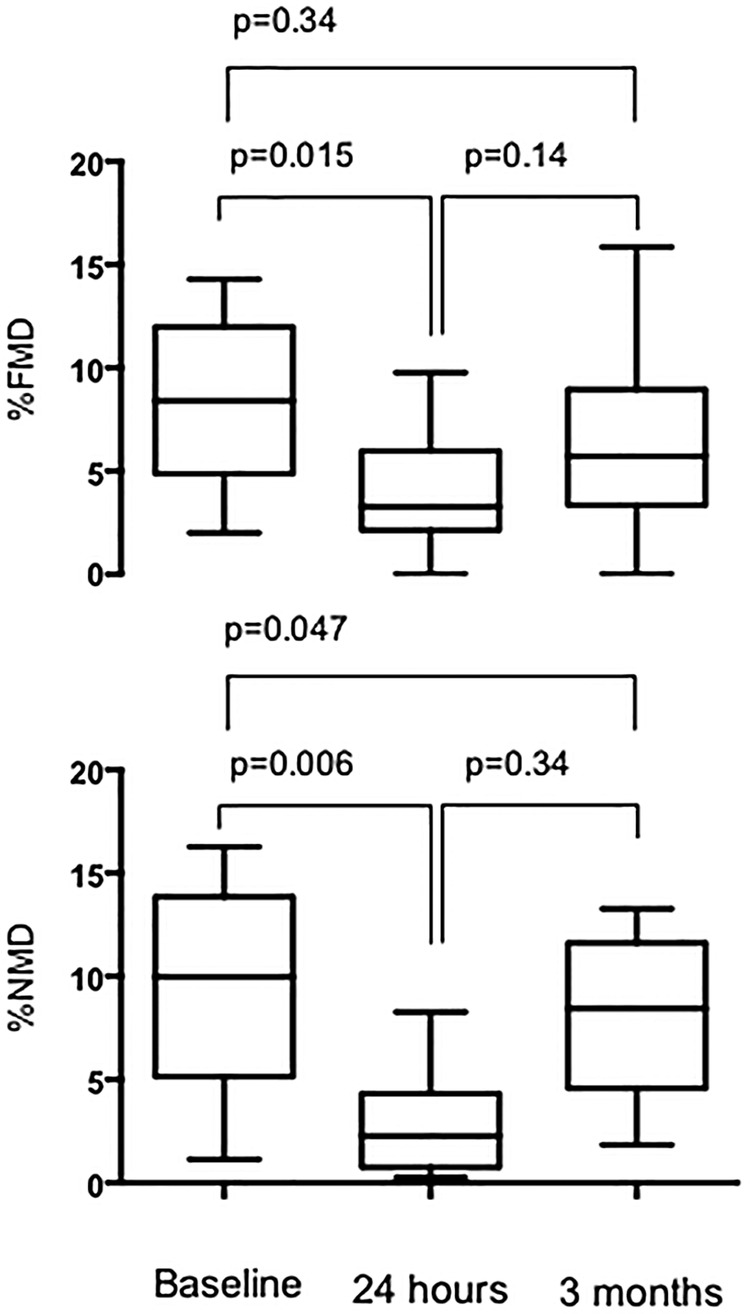
Flow-mediated and nitrate-mediated dilation in following radial artery catheterisation. Flow-mediated dilation (FMD) in the catheterised right radial artery is impaired at 24 hours (3.9±2.9%) when compared to baseline (8.6±4.0%, p=0.015). There was some degree of functional recovery by 3 months (6.2±4.4%) by which time FMD was not significantly different to baseline (p=0.34). Nitrate-mediated dilation (NMD) was similarly impaired at 24 hours (2.8±2.5%) when compared to baseline (9.4±5.1%, p=0.006). Again, there was some improvement by 3 months (4.8±2.7%) but this had not returned to baseline (p=0.047).

In the 10 patients undergoing cardiac catheterisation, baseline radial diameter was 2.8±0.5 mm (table 1). Compared with baseline, radial artery FMD was impaired at 24 hours (8.6±4.0% vs 3.9±2.9%, p=0.015) but not at 3 months (8.6±4.0% vs 6.2±4.4%, p=0.34; figure 6) following transradial catheterisation.

As with FMD, compared with baseline NMD was impaired at 24 hours (8.7±5.5% vs 2.8±2.5%, p=0.006). Response to nitrates remained impaired at 3 months (8.7±5.5% vs 4.8±2.7%, p=0.047; figure 6) following transradial catheterisation.

## Discussion

We have here demonstrated that FMD and NMD can be used to assess radial artery vasomotor function in man with acceptable reproducibility. Using this technique, we have demonstrated that radial artery vasomotor function is impaired early after transradial access for cardiac catheterisation, but appears to recover, at least in part, by 3 months with endothelium-dependent vasodilatation no longer significantly different from baseline. While impairment of radial function postcatheterisation has previously been described, reproducibility of this technique is not well characterised; adding to the work of Brook *et al*^18^ who characterised reproducibility in healthy volunteers, we have also made an assessment in a patient cohort. Our findings suggest that transradial access for cardiac catheterisation may afford a potential model of vascular injury and repair in vivo in man.

Previous studies have demonstrated that the heterogeneity observed in the FMD response is dependent in part on the flow stimulus^19^ ^20^ as well as underlying physical characteristics of participants.^19^ To address these potential concerns, we standardised the environmental conditions and experimental protocol, although we did not document the flow stimulus by Doppler ultrasound. We observed minimal within-day and between-day variability in the measurement of absolute radial artery diameter using ultrasound. However, we did observe more prominent heterogeneity in the relative responses to FMD and NMD in healthy volunteers and patients.

The variability in radial FMD is more pronounced than the more widely used brachial FMD, for which a coefficient of variation of 1.8% has previously been reported in the original study by Sorensen *et al*.^21^ However, the calculation of this variability was unclear, and while subsequent studies have reported that baseline brachial artery diameter can be measured reproducibly, most report a coefficient of variation for brachial FMD of between 10% and 50%.^22–25^

Although the resting diameter of the radial artery is smaller than the brachial artery, vasodilatation causes similar the absolute increases in arterial diameter with both arteries. This has some important effects on the calculation of the relative diameter changes for FMD and NMD. In other words, the percentage change in vessel size is larger for radial than brachial FMD because of the smaller denominator. While superficially this is attractive for the detection of changes due to therapeutic interventions, inaccuracies will be exaggerated and reproducibility compromised. The lack of linearity between baseline diameter and absolute FMD response has been suggested as a confounder in comparisons between and within participants. Atkinson *et al*^26^ suggest a promising way of correcting for this, although this is yet to be widely adopted. Ultimately our data demonstrate that absolute and relative changes in radial FMD and NMD are more variable than those seen for the brachial artery and this is consistent with previous work.^18^

Dilation of the radial artery in the human forearm in response to short periods of ischaemia is dependent on an intact endothelium and is attenuated by infusion of the nitric oxide synthase inhibitor, *N*^G^monomethyl-L-arginine.^19^ Acute disruption of the vessel wall has been demonstrated following transradial cardiac catheterisation using optical coherence tomography-based intravascular imaging.^9^ ^10^ The catheterised radial artery has also been examined histologically at the time of conduit harvest for coronary artery bypass grafting with one study demonstrating endothelial disruption, with the degree of endothelial loss inversely related to the time since catheterisation,^27^ although this has not been a universal finding.^28^ It is perhaps not surprising therefore that we and others^12–14^ ^29–31^ have demonstrated that transradial catheterisation results in impairment of FMD of the radial artery. This likely represents endothelial denudation with recovery of vasomotor function indicating reconstitution of the monolayer. The attenuation of FMD and NMD after radial catheterisation is in keeping with previous studies^13^ ^14^ and implies that the vascular injury involves the vascular smooth muscle layer and the superficial endothelial layer. The time course of recovery remains unclear with some authors reporting complete recovery of radial artery vasomotor function,^11^ ^29^ ^32^ while others observed irreversible impairment.^14^ Our data would suggest that there is some recovery of function at 3 months, but we cannot be confident of complete restoration of function.

Previous studies have used the radial artery in the context of cardiac catheterisation to examine the influence of factors such as sheath coating^13^ and drug therapy^2^ ^31^ ^33^ on endothelial recovery. These models have however only examined forearm vasomotion and at a limited number of time points—a limitation shared by our study. Understanding the exact mechanism, the time course of injury and its recovery is critical if this model is to realise its potential in the study of vascular injury and repair in vivo in man.

It is important to highlight a number of limitations of our study. In addition to the modest sample size and innate heterogeneity observed in forearm vasomotor responses, it would be helpful to include the contralateral arm as control in any future studies examining this model in more detail. While our protocol was standardised to minimise variation in hyperaemic stimulus, we did not quantitatively assess radial blood flow velocity, variations in which may have affected the vasodilator response. We also did not attempt to characterise the extent or mechanism of injury inflicted on the radial artery at the time of catheterisation. This could have been achieved with intravascular imaging modalities such as intravascular ultrasound or optical coherence tomography. Further work, combining intravascular imaging with cellular and cytokine profiling, would define this in greater detail and improve our understanding of this promising model of in vivo arterial injury in man.

## Conclusion

While radial FMD is a technique with inherent variability, the larger magnitude of baseline response than brachial FMD and the profound impact of catheterisation on endothelial function mean that the effect of arterial injury can be demonstrated in a small cohort of patients. Combined with the ubiquitous use of radial access and the non-invasive nature of FMD, this makes transradial cardiac catheterisation a powerful and accessible tool for studying mechanical vascular injury. We believe that this model will permit mechanistic insights into the processes of vascular injury and repair as well as the modifying influences of cardiovascular risk factors and therapies.

## References

[1] Hackam DG, Redelmeier DA Translation of research evidence from animals to humans. JAMA 2006;296:1731–2. 10.1001/jama.296.14.1731 17032985

[2] Li JM, Eslami MH, Rohrer MJ Interleukin 18 binding protein (IL18-BP) inhibits neointimal hyperplasia after balloon injury in an atherosclerotic rabbit model. J Vasc Surg 2008;47:1048–57. 10.1016/j.jvs.2007.12.005 18455646

[3] Tanaka K, Sata M Contribution of circulating vascular progenitors in lesion formation and vascular healing: lessons from animal models. Curr Opin Lipidol 2008;19:498–504. 10.1097/MOL.0b013e32830dd566 18769231

[4] Silvestre JS, Mallat Z, Duriez M Antiangiogenic effect of interleukin-10 in ischemia-induced angiogenesis in mice hindlimb. Circ Res 2000;87:448–52. 10.1161/01.RES.87.6.448 10988235

[5] Jacobi J, Tam BYY, Wu G Adenoviral gene transfer with soluble vascular endothelial growth factor receptors impairs angiogenesis and perfusion in a murine model of hindlimb ischemia. Circulation 2004;110:2424–9. 10.1161/01.CIR.0000145142.85645.EA 15477417

[6] Moore S, Belbeck LW, Richardson M Lipid accumulation in the neointima formed in normally fed rabbits in response to one or six removals of the aortic endothelium. Lab Invest 1982;47:37–42. 7087396

[7] Strauss BH, Chisholm RJ, Keeley FW Extracellular matrix remodeling after balloon angioplasty injury in a rabbit model of restenosis. Circ Res 1994;75:650–8. 10.1161/01.RES.75.4.650 7923611

[8] Rodriguez-Menocal L, St-Pierre M, Wei Y The origin of post-injury neointimal cells in the rat balloon injury model. Cardiovasc Res 2009;81:46–53. 10.1093/cvr/cvn265 18818213

[9] Yonetsu T, Kakuta T, Lee T Assessment of acute injuries and chronic intimal thickening of the radial artery after transradial coronary intervention by optical coherence tomography. Eur Heart J 2010;31:1608–15. 10.1093/eurheartj/ehq102 20413398

[10] Di Vito L, Burzotta F, Trani C Radial artery complications occurring after transradial coronary procedures using long hydrophilic-coated introducer sheath: a frequency domain-optical coherence tomography study. Int J Cardiovasc Imaging 2013;30:21–9. 10.1007/s10554-013-0284-9 24154615

[11] Yan Z, Zhou Y, Zhao Y Impact of transradial coronary procedures on radial artery function. Angiology 2010;61:8–13. 10.1177/0003319709348293 19815606

[12] Dawson EA, Rathore S, Cable NT Impact of catheter insertion using the radial approach on vasodilatation in humans. Clin Sci 2010;118:633–40. 10.1042/CS20090548 20059449

[13] Dawson EA, Rathore S, Cable NT Impact of introducer sheath coating on endothelial function in humans after transradial coronary procedures. Circ Cardiovasc Interventions 2010;3:148–56. 10.1161/CIRCINTERVENTIONS.109.912022 20407113

[14] Burstein JM, Gidrewicz D, Hutchison SJ Impact of radial artery cannulation for coronary angiography and angioplasty on radial artery function. Am J Cardiol 2007;99:457–9. 10.1016/j.amjcard.2006.08.055 17293183

[15] Dawson EA, Alkarmi A, Thijssen DHJ Low-flow mediated constriction is endothelium-dependent: effects of exercise training after radial artery catheterization. Circ Cardiovasc Interv 2012;5:713–19. 10.1161/CIRCINTERVENTIONS.112.971556 23011264

[16] Corretti M, Anderson T, Benjamin E Guidelines for the ultrasound assessment of endothelial-dependent flow-mediated vasodilation of the brachial artery: a report of the International Brachial Artery Reactivity Task Force. J Am Coll Cardiol 2002;39:257–65. 11788217 10.1016/s0735-1097(01)01746-6

[17] Thijssen DHJ, Black MA, Pyke KE Assessment of flow-mediated dilation in humans: a methodological and physiological guideline. Am J Physiol Heart Circ Physiol 2011;300:H2–12. 10.1152/ajpheart.00471.2010 20952670 PMC3023245

[18] Brook R, Grau M, Kehrer C Intrasubject variability of radial artery flow-mediated dilatation in healthy subjects and implications for use in prospective clinical trials. Am J Cardiol 2005;96:1345–8. 10.1016/j.amjcard.2005.06.086 16253612

[19] Mullen MJ, Kharbanda RK, Cross J Heterogenous nature of flow-mediated dilatation in human conduit arteries in vivo: relevance to endothelial dysfunction in hypercholesterolemia. Circ Res 2001;88:145–51. 10.1161/01.RES.88.2.145 11157665

[20] Agewall S, Doughty RN, Bagg W Comparison of ultrasound assessment of flow-mediated dilatation in the radial and brachial artery with upper and forearm cuff positions. Clin Physiol 2001;21:9–14. 11168291 10.1046/j.1365-2281.2001.00302.x

[21] Sorensen KE, Celermajer DS, Spiegelhalter DJ Non-invasive measurement of human endothelium dependent arterial responses: accuracy and reproducibility. Br Heart J 1995;74:247–53. 10.1136/hrt.74.3.247 7547018 PMC484014

[22] Liang YL, Reede H, Kotsopoulos D Non-invasive measurements of arterial structure and function: repeatability, interrelationships and trial sample size. Clin Sci 1998;95:669–79. 10.1042/cs0950669 9831691

[23] De Roos NM, Bots ML, Katan MB Replacement of dietary saturated fatty acids by trans fatty acids lowers serum HDL cholesterol and impairs endothelial function in healthy men and women. Arterioscler Thromb Vasc Biol 2001;21:1233–7. 10.1161/hq0701.092161 11451757

[24] De Roos NM, Bots ML, Katan MB Within-subject variability of flow-mediated vasodilation of the brachial artery in healthy men and women: implications for experimental studies. Ultrasound Med Biol 2003;29:401–6. 10.1016/S0301-5629(02)00709-3 12706191

[25] Hijmering ML, Stroes ES, Pasterkamp G Variability of flow mediated dilation: consequences for clinical application. Atherosclerosis 2001;157:369–73. 10.1016/S0021-9150(00)00748-6 11472736

[26] Atkinson G, Batterham AM, Thijssen DHJ A new approach to improve the specificity of flow-mediated dilation for indicating endothelial function in cardiovascular research. J Hypertens 2013;31:287–91. 10.1097/HJH.0b013e32835b8164 23169234

[27] Kamiya H, Ushijima T, Kanamori T Use of the radial artery graft after transradial catheterisation: is it suitable as a bypass conduit? Ann Thorac Surg 2003;76:1505–9. 10.1016/S0003-4975(03)01018-X 14602276

[28] Gaudino M, Leone A, Lupascu A Morphological and functional consequences of transradial coronary angiography on the radial artery: implications for its use as a bypass conduit. Eur J Cardiothorac Surg 2015;48:370–4. 10.1093/ejcts/ezu456 25475945

[29] Madssen E, Haere P, Wiseth R Radial artery diameter and vasodilatory properties after transradial coronary angiography. Ann Thorac Surg 2006;82:1698–702. 10.1016/j.athoracsur.2006.06.017 17062231

[30] Okuyan H, Açikgoz SK, Tacoy G Effect of transradial coronary angiography procedure on vascular diameter and vasodilator functions in the access site. Angiology 2012;64: 515–21. 10.1177/0003319712458450 22949740

[31] Park KH, Park DK, Kim MK Effects of sheath injury and Trimetazidine on endothelial dysfunction of radial artery after transradial catheterisation. J Interv Cardiol 2012;25:411–17. 10.1111/j.1540-8183.2012.00735.x 22536918

[32] Tilling L, Hunt J, Donald A Arterial injury and endothelial repair: rapid recovery of function after mechanical injury in healthy volunteers. Cardiol Res Pract 2014;2014:367537 10.1155/2014/367537 24719774 PMC3956421

[33] Turan B, Erkol A, Yilmaz F Incidence and predictors of radial artery injury following transradial procedures: yet another benefit of renin-angiotensin system blockade? Cardiol J 2015;23:64–70. 26412611 10.5603/CJ.a2015.0057

